# Extended Grunwald-Winstein Analysis - LFER Used to Gauge Solvent Effects in *p-*Nitrophenyl Chloroformate Solvolysis

**DOI:** 10.3390/ijms9112231

**Published:** 2008-11-13

**Authors:** Malcolm J. D’Souza, Kevin E. Shuman, Shannon E. Carter, Dennis N. Kevill

**Affiliations:** 1 Department of Chemistry, Wesley College, 120 N. State Street, Dover, Delaware 19901-3875; 2 Department of Chemistry and Biochemistry, Northern Illinois University, DeKalb, Illinois 60115-2862, USA

**Keywords:** Solvolysis, addition-elimination, association-dissociation, *p*-nitrophenyl chloroformate, chloroformates, Grunwald-Winstein equation, LFERS, leaving group effects

## Abstract

Specific rates of solvolysis at 25 °C for *p*-nitrophenyl chloroformate (**1**) are analyzed using the extended (two-term) Grunwald-Winstein equation. For 39 solvents, the sensitivities (*l* = 1.68±0.06 and *m* = 0.46±0.04) towards changes in solvent nucleophilicity (*l*) and solvent ionizing power (*m*) obtained, are similar to those previously observed for phenyl chloroformate (**2**) and *p*-methoxyphenyl chloroformate (**3**). The observations incorporating new kinetic data in several fluoroalcohol-containing mixtures, are rationalized in terms of the reaction being sensitive to substituent effects and the mechanism of reaction involving the addition (association) step of an addition-elimination (association-dissociation) pathway being rate-determining. The *l*/*m* ratios obtained for **1**, **2**, and **3**, are also compared to the previously published *l*/*m* ratios for benzyl chloroformate (**4**) and *p-*nitrobenzyl chloroformate (**5**).

## 1. Introduction

**Figure 1.**
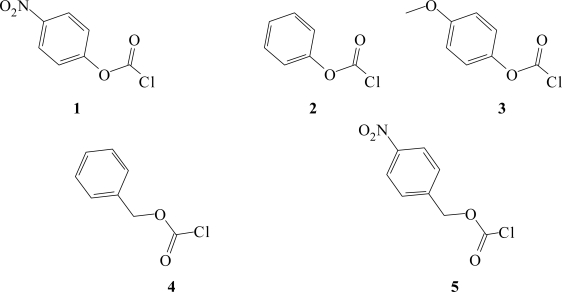
Molecular structures of *p-*nitrophenyl chloroformate (**1**), phenyl chloroformate (**2**), *p-*methoxyphenyl chloroformate (**3**), benzyl chloroformate (**4**), and *p-*nitrobenzyl chloroformate (**5**).

Chloroformates (Figure 1) [1, 2] have been extensively used to study substitution reactions involving the replacement of chlorine to an acyl carbon, as acid chlorides of the type RCOCl tend to react too fast for the kinetics to be followed by conventional techniques [3, 4]. An early survey [5] of the kinetics of solvolytic reactions including hydrolysis, suggested that nucleophilic substitution reactions of chloroformates [RO(CO)Cl] formally parallel those of other types of carboxylic acid esters. Due to an increased initial state stabilization [5–9] experienced with the insertion of an oxygen atom between the R group and the acyl carbon, it is possible to obtain information on the kinetics of the solvolysis process of a number of alkyl and aryl chloroformate esters by standard titrimetric methods.

**Scheme 1.**
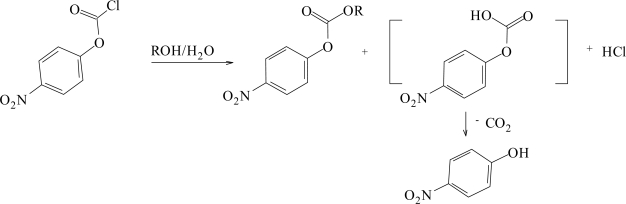
Alcoholysis of *p-*nitrophenyl chloroformate (**1**).

Chloroformate esters such as *p*-nitrophenyl chloroformate (**1**) have found increased use in the synthesis of aromatic polycarbonates for biomaterials [10], and in the present contribution its solvolyses expressed in Scheme 1, were conveniently followed at 25.0°C in ten fluoroalcohol containing solvents.

Sixty years ago the original Grunwald-Winstein equation (equation 1) was proposed [11] for the correlation of specific rates of solvolysis of initially neutral substrates reacting by an ionization (S_N_1 + E1) mechanism:

(1) $$\text{log}\;(k/k_{0})=mY+c$$

In equation (1), *k* and *k_o_* are the specific rates of solvolysis in a given solvent and in the standard solvent (80% ethanol), respectively, *m* represents the sensitivity to changes in the solvent ionizing power *Y* (initially set at unity for *tert*-butyl chloride solvolyses), and *c* is a constant (residual) term. It is now realized both that the scales are leaving-group dependent and that adamantyl derivatives provide better standard substrates, and for a leaving group X a series of *Y_X_* scales are available [12].

For bimolecular (S_N_2 and/or E2) reactions [13], the correlation is extended (equation 2) to include a term governed by the sensitivity *l* to changes in solvent nucleophilicity (*N*):

(2) $$\text{log}\;(k/k_{0})=lN+mY+c$$

Initially, Schadt, Bentley, and Schleyer [14] used methyl *p*-toluenesulfonate, to arrive at the *N_OTs_* scale. More recently *N_T_* scales based on the solvolyses of *S*-methyldibenzothiophenium ion [15] have been developed, in which the leaving group is a neutral molecule, which is little influenced by solvent change, and these values [15, 16] have become the recognized standards for considerations of solvent nucleophilicity. A problem with multiparameter equations, such as equation (2), is that strong covariances [17] are often observed between *N* and *Y* values, hence, Bentley’s group [18] favors the use of equation (1) rather than equation (2) in looking for weak nucleophilic assistance, because they claim that when multiparameter equations are employed, novel effects may not be detected because of the tendency to correlate all of the data moderately successfully. A recent review [19] from our laboratories, examined the development and uses of extended forms of the Grunwald-Winstein equation in a much greater detail than is presented in this manuscript.

During the past two decades, the Grunwald-Winstein equations (equations 1, 2) have emerged as powerful mechanistic tools that are utilized to understand solvolysis mechanisms in alkyl [20–28], alkenyl [29], and aryl [30–37] chloroformate esters. The published results corroborate recent suggestions [9, 19, 38, 39] that acid chlorides of monoesters of carbonic acid and of carboxylic acids tend to solvolyze with competing addition-elimination (with rate-determining addition) and ionization S_N_1 (assisted by nucleophilic solvation) pathways. The extended form of the Grunwald-Winstein equation (equation 2) has been applied successfully to the specific rates of solvolysis of phenyl chloroformate (**2**) [30, 36] and *p-*methoxyphenyl chloroformate (**3**) [36, 37] over the full range of solvents employed in these types of LFER studies. The reported [37] *l* value (bond making) of 1.60±0.05 and *m* value (bond breaking) of 0.57±0.05 for **2**; and a value of 1.66±0.05 for *l*, and a *m* value of 0.56±0.03 for **3** [36, 37], are consistent with what one would expect for the addition step of an addition-elimination mechanism being rate-determining. In the present study, we have augmented previously published [32, 33] specific rates of solvolyses of *p-*nitrophenyl chloroformate (**1**) in order to analyze the contributions made by the excellent electron withdrawing nitro group which makes the carbonyl carbon more positive and thus more susceptible to nucleophilic attack. To better determine whether the nitro group manifests this electron withdrawing character we have extended the prior study [32, 33] by adding additional solvents having an appreciable fluoroalcohol component. Fluoroalchols have been shown to be extremely important, either as pure solvents or as components of binary mixtures, in studies leading to analyses in terms of Grunwald-Winstein equations [40–42].

## 2. Results and Discussion

To give additional specific rates of solvolysis of *p-*nitrophenyl chloroformate (**1**), five values in aqueous 2,2,2-trifluoroethanol (TFE), three values in aqueous 1,1,1,3,3,3-hexafluoro-2-propanol (HFIP), and two values in TFE-ethanol mixtures were measured at 25.0 °C. These specific rate values are reported in Table 1.

**Figure 2.**
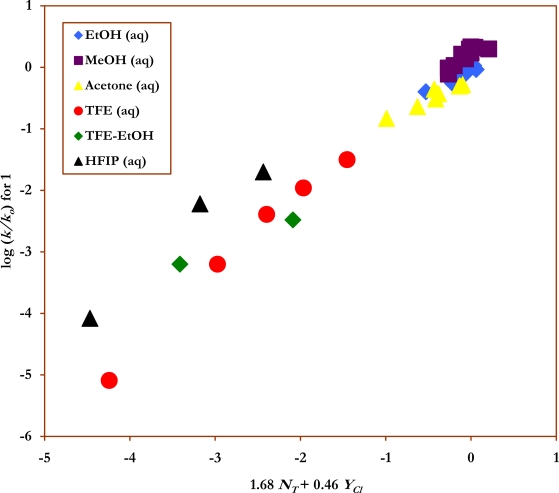
The plot of log (*k/k_o_*) vs. (1.68 *N_T_* + 0.46 *Y_Cl_*) for the solvolyses of *p-*nitrophenyl chloroformate (**1)** in pure and binary solvents at 25.0 °C.

**Scheme 2.**
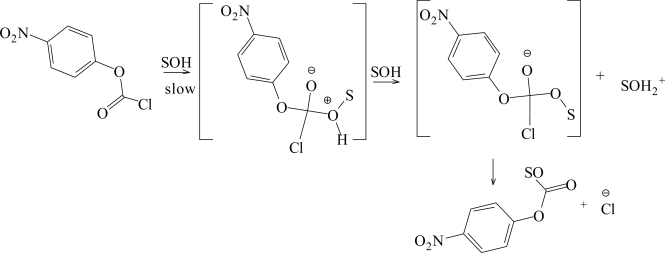
Stepwise addition-elimination mechanism through a tetrahedral intermediate proposed for *p-*nitrophenyl chloroformate (**1**).

For the solvents listed in Table 1, the nitro group exerts a greater inductive electron withdrawing effect making the nucleophilic solvent attack at the electrophlic carbonyl carbon center easier, and accordingly the reported specific rates of solvolysis increase in the order; *k* **(1)** > *k* **(2)** ≈ *k* (**3),** indicating that a bimolecular type mechanism is in operation. In Table 2, the initial sensitivity values were obtained (*l* = 1.85±0.21 and *m* = 0.48±0.05) from the correlation analyses using just the previously studied data of 29 aqueous ethanol, aqueous methanol, and aqueous acetone mixtures [32, 33], together with *N_T_* values [16] and *Y_Cl_* values [42–44]. This multiple regression analysis using the extended Grunwald-Winstein equation (equation 2), gave a multiple correlation coefficient of 0.870, and an *F* test value of 41, suggesting that these results must be interpreted with considerable caution.The new data in ten solvents with appreciable fluoroalcohol content (Table 1) were then combined with the 29 literature values [32, 33]. For the 39 solvents, we obtained a good linear correlation with values of *l* = 1.68±0.06, *m* = 0.46±0.04, *c* = 0.074±0.08, 0.976 for the correlation coefficient, and 363 for the *F-*test value (Figure 2). With the application of equation 2, both the multiple correlation coefficient (from 0.870 to 0.976) and the *F-*test values (from 41 to 363), improved considerably on inclusion of the 10 fluoroalcohol data points from Table 1. These improvements illustrate the need for a good selection of solvents for a meaningful application of extended form of the Grunwald-Winstein equation. The observed *l* and *m* values are within the range previously observed for other reactions at acyl carbon which are believed to proceed by an addition-elimination (association-dissociation) mechanism shown in Scheme 2, with the addition step rate-determining [19, 20, 22–25, 27–39].

For a meaningful comparison of the extended Grunwald-Winstein results for the specific rates of solvolysis of **1**, **2**, and **3**, at 25.0 °C reported in Table 2, it is important that the comparison is made in identical solvents. For 38 common solvents (90T-10E was excluded), results obtained are *l* = 1.69±0.07, *m* = 0.46±0.04, *c* = 0.074±0.08, *R* = 0.974, *F-*test = 323, for **1**; *l* = 1.59±0.07, *m* = 0.54±0.03, *c* = 0.16±0.08, *R* = 0.972, *F-*test = 299, for **2**; and *l* = 1.58±0.06, *m* = 0.57±0.04, *c* = 0.17±0.07, *R* = 0.974, F-test = 320, for **3**. The *l*/*m* ratios decreased from a value of 3.67 for **1**, to 2.94 for **2**, and to 2.77 for **3**. This trend implies an earlier transition state for the rate-determining addition step in **1**, and the importance of general base catalysis decreases in going from **1**, to **2** and **3**. The importance of general-base catalysis in solvolyses of aryl chloroformates is indicated by large kinetic solvent isotope effects (KSIEs) in methanol and methanol-*d* (*k*_MeOH_/*k*_MeOD_). Unfortunately, there is an ambiguity regarding the value for *p*-nitrophenyl chloroformate, with values of 2.51±0.07 [45] and 2.10 [33] having been reported (the difference arises from a discrepancy between the *k*_MeOD_ values). In one study, the value is higher than for other aryl chloroformates and, in the other, it is lower. Either value suggests, however, that general base catalysis is operating [28].

**Figure 3.**
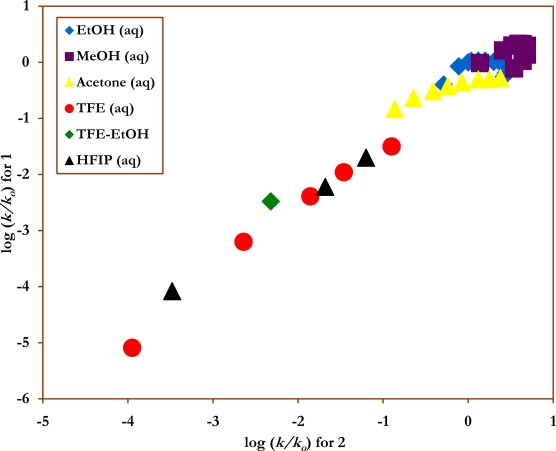
The plot of log (*k/k_o_*) for *p-*nitrophenyl chloroformate (**1**) against log (*k/k_o_*) for phenyl chloroformate (**2**) in pure and binary solvents at 25.0 °C.

The *l/m* ratios of 2.96 for **2** and 2.80 for **3** previously reported [37] in 44 solvents, are very close to the values of 2.94 for **2**, to 2.77 for **3** reported in Table 2 above for 38 solvents. The *l/m* ratios are important in two regards. They compensate for earlier and later transition states within otherwise very similar mechanisms and, if one is carrying out a *direct* logarithmic correlation of the specific rates for two compounds for which both solvent nucleophilicity and ionizing power effects are important, the requirement for a good linear correlation is a close similarity in the *l/m* ratios for the two substrates. For example, the similarity in *l*/*m* ratios for **2** and **3** suggest that a very good direct linear relationship exists between their specific rates of solvolysis, which was indeed the case in the excellent linear plot [37] of log (*k/k_o_*) values for **3** against those for **2** with a correlation coefficient of 0.998, *F-*test value of 9302, slope of 0.991±0.010, and intercept of 0.075±0.015. The larger differences in the *l/m* ratios of 3.67 for **1** and 2.96 for **2**, are substantiated by the plot shown in Figure 3 of log (*k/k_o_*) values for **1** against those for **2**, with a reduced correlation coefficient of 0.981, *F-*test value of 933, slope of 1.07±0.04, and intercept of –0.35±0.04.

For the 15 solvolyses considered [35] to be via addition-elimination for benzyl chloroformate (**4**), values were obtained of 1.95±0.16 for *l*, 0.57±0.05 for *m*, 0.16±0.15 for *c*, *R* of 0.966 and *F-*test value of 83. The *l*/*m* value reported of 3.42 for these 15 solvents, was essentially identical to the value of 3.50 obtained for *p-*nitrobenzyl chloroformate (**5**) in 19 solvents [35], where values of 1.61±0.09 for *l*, 0.46±0.04 for *m*, 0.04±0.22 for *c*, *R* of 0.975 and *F-*test value of 157 were obtained. The identical *l*/*m* ratios suggest the presence of a tighter rate-determining tetrahedral transition state, and that the importance of general base catalysis is very similar for benzyl and *p-*nitrobenzyl chloroformate esters.

**Figure 4.**
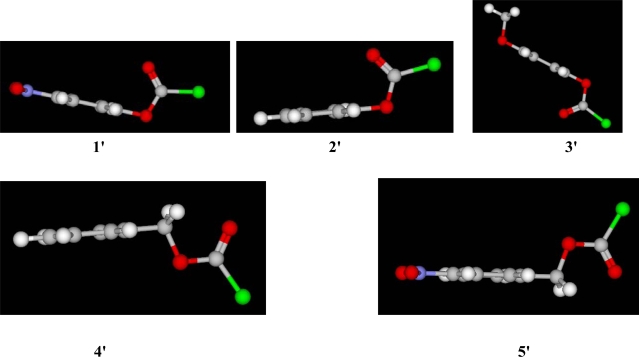
3-D views for *p-*nitrophenyl chloroformate (**1**′), phenyl chloroformate (**2′**), *p*-methoxyphenyl chloroformate (**3′**), benzyl chloroformate (**4**′), and *p-*nitrobenzyl chloroformate (**5**′), computed using the KnowItAll® platform.

A visual inspection of the computed 3-D views shown in Figure 4, reveals the reason of the powerful inductive effect observed in **1**. The planarity observed with the *p-*nitrophenyl group and its ether oxygen as shown in **1′** makes them linear, so that they can exert their full inductive ability, and this further substantiates the differences seen in the *l/m* ratios reported in Table 1 for **1**, **2**, and **3**. The specific rate order [this work, 33–35] of *k* **(1)** > *k* **(5)** in all of the common solvents studied, is probably in part because the *p-*nitrobenzyl group twists out of the plane with its ether oxygen (as shown in **5′**), and therefore is able to exert only a fraction of its possible inductive capability.

## 3. Conclusions

The extended Grunwald-Winstein equation is a versatile tool that can be effectively used to gauge solvent effects in solvolysis reactions. The presently reported analyses demonstrate that meaningful contributions associated with the extended Grunwald-Winstein treatment of **1** can result when an adequate selection of solvents with considerably different *N_T_* and *Y_Cl_* values are made available. The *l* and *m* parameters obtained for **1** are very similar to other chloroformate esters (such as **2**, and **3**) where the addition step of an addition-elimination pathway is rate-determining. The trends in the *l*/*m* ratios observed (Table 2) and observations from the 3D-views shown above, support our proposal of an earlier step-wise transition state for **1**, and a decrease in the importance of general base catalysis in going from **1** to **2** and **3**.

## 4. Experimental Section

The *p-*nitrophenyl chloroformate (Sigma-Aldrich, 96%) was used as received. Solvents were purified and the kinetic runs carried out as described previously [41]. A 0.6 <u>M</u> stock solution was made in acetonitrile (Sigma-Aldrich, 99.5%) and a substrate concentration of approximately 0.03 <u>M</u> in a variety of fluoroalcohols was employed. The calculation of the specific rates of solvolysis (first-order rate coefficients) were obtained when the conventional Guggenheim treatment [46] was modified [47] so as to give the infinity titer, which was then used to calculate for each run a series of integrated rate coefficients. The specific rates and associated standard deviations, as presented in Table 1, are obtained by averaging all of the values from, at least, duplicate runs.

Multiple regression analysis were carried out using the Excel 2007 package from the Microsoft Corporation, and the SigmaPlot 9.0 software version from Systat Software, Inc., San Jose, CA, was used for the Guggenheim treatments. Incorporating prior results [9] for the assigned position of the halogen in the ground-state structure of **2**, the 3D-views presented in Figure 4 for the 5 molecules in this study were computed using the KnowItAll® Informatics System, ADME/Tox Edition, from Bio-Rad Laboratories, Philadelphia, PA. The KnowItAll® platform contains a 3-D molecular rendering program SymApps™ that uses a modified MM2 force field minimization module to convert 2-D structure drawings to 3-D images.

## Figures and Tables

**Table 1.** Specific rates of solvolysis (*k*) of *p-*nitrophenyl chloroformate (**1**), phenyl chloroformate (**2**), and *p-*methoxyphenyl chloroformate (**3**), in several binary solvents at 25.0°C and the solvent nucleophilicity (*N_T_*), and the solvent ionizing power (*Y_Cl_*) values, for the solvents.

Solvent (%)*^a^*	1; 10^5^*k*(s^−1^)*^b^*	2; 10^5^*k*(s^−1^)*^c^*	3; 10^5^*k*(s^−1^)*^d^*	$N_{T}^{e}$	$Y_{Cl}^{f}$
97% TFE (w/w)	0.113±0.008	0.0570±0.0030	0.0300±0.0013	−3.30	2.83
90% TFE (w/w)	8.87±0.28	1.15±0.08	0.825±0.032	−2.55	2.85
80% TFE (w/w)	56.8±0.4	7.02±0.28	8.63±0.24	−2.22	2.90
70% TFE (w/w)	153±1.5	17.4±1.3	15.2±0.6	−1.98	2.96
50% TFE (w/w)	438±44	63.5±3.0	52.6±2.8	−1.73	3.16
90T-10E (v/v)	8.82±0.17			−2.67	2.33
80T-20E (v/v)	45.5±0.7	2.43±0.21	3.52±0.13	−1.76	1.89
90%HFIP (w/w)	1.20±0.06	0.166±0.004	0.172±0.007	−3.84	4.31
70%HFIP (w/w)	83.8±0.9	10.5±0.3	7.58±0.22	−2.94	3.83
50%HFIP (w/w)	277±2	31.6±0.6	24.9±0.5	−2.49	3.80

*^a^* Volume-volume (v/v) basis at 25.0°C or weight-weight (w/w) basis, as described; other component water, except for TFE-ethanol (T-E) solvents.

*^b^* with associated standard deviation.

*c* From refs. [30, 36].

*^d^* From refs. [30, 36, 37].

*^e^* From ref. [16].

*^f^* From refs. [42–44].

**Table 2.** Correlations*^a^* of the specific rates of solvolyses of **1**, and a comparison with the corresponding specific rate values for **2** and **3** in identical solvents.

Substrate	*n^b^*	*ℓ^c^*	*m^c^*	*c^c^*	*l/m*	*R^d^*	*F^e^*
	29^f^	1.85±0.21	0.48±0.05	0.14±0.05	3.85	0.871	41
**1**	39^g^	1.68±0.06	0.46±0.04	0.074±0.08	3.65	0.976	363
	38^h^	1.69±0.07	0.46±0.04	0.074±0.08	3.67	0.974	323
**2**	38^h^	1.59±0.07	0.54±0.03	0.16±0.08	2.94	0.972	299
**3**	38^h^	1.58±0.06	0.57±0.04	0.17±0.07	2.77	0.974	320

*^a^* Using equation (2).

*^b^* Number of data points.

*^c^* With associated standard error.

*^d^* Correlation coefficient. *^e^* *F*-test value.

*^f^* Specific rates are from the 29 solvents reported in refs. [32, 33].

*g* Specific rates are those from Table 1 plus the 29 used in refs. [32, 33].

*^h^* Without 90T-10E; using identical solvents for the 38 data-point correlation of the specific rates of solvolysis of **1**, **2**, and **3**.
